# Targeting the Complement Serine Protease MASP-2 as a Therapeutic Strategy for Coronavirus Infections

**DOI:** 10.3390/v13020312

**Published:** 2021-02-17

**Authors:** Ben M. Flude, Giulio Nannetti, Paige Mitchell, Nina Compton, Chloe Richards, Meike Heurich, Andrea Brancale, Salvatore Ferla, Marcella Bassetto

**Affiliations:** 1 Department of Chemistry, College of Science and Engineering, Swansea University, Swansea SA28PP, UK; 959957@Swansea.ac.uk (B.M.F.); 955243@Swansea.ac.uk (P.M.); marcella.bassetto@swansea.ac.uk (M.B.); 2 School of Pharmacy and Pharmaceutical Sciences, Cardiff University, Cardiff CF10 3NB, UK; nannettig@cardiff.ac.uk (G.N.); heurichm@cardiff.ac.uk (M.H.); 3 Crosskeys Campus, Coleg Gwent, Crosskeys NP117ZA, UK; marcellabassetto@gmail.com; 4 Ysgol Gyfun Rhydywaun, Rhondda Cynon Taf CF449ES, UK; benedetta.bassetto@gmail.com; 5 Swansea University Medical School, Swansea SA28PP, UK; salvatore.ferla@swansea.ac.uk

**Keywords:** MASP-2, coronaviruses, molecular modelling, drug repurposing

## Abstract

MASP-2, mannose-binding protein-associated serine protease 2, is a key enzyme in the lectin pathway of complement activation. Hyperactivation of this protein by human coronaviruses SARS-CoV, MERS-CoV and SARS-CoV-2 has been found to contribute to aberrant complement activation in patients, leading to aggravated lung injury with potentially fatal consequences. This hyperactivation is triggered in the lungs through a conserved, direct interaction between MASP-2 and coronavirus nucleocapsid (N) proteins. Blocking this interaction with monoclonal antibodies and interfering directly with the catalytic activity of MASP-2, have been found to alleviate coronavirus-induced lung injury both in vitro and in vivo. In this study, a virtual library of 8736 licensed drugs and clinical agents has been screened in silico according to two parallel strategies. The first strategy aims at identifying direct inhibitors of MASP-2 catalytic activity, while the second strategy focusses on finding protein-protein interaction inhibitors (PPIs) of MASP-2 and coronaviral N proteins. Such agents could represent promising support treatment options to prevent lung injury and reduce mortality rates of infections caused by both present and future-emerging coronaviruses. Forty-six drug repurposing candidates were purchased and, for the ones selected as potential direct inhibitors of MASP-2, a preliminary in vitro assay was conducted to assess their interference with the lectin pathway of complement activation. Some of the tested agents displayed a dose-response inhibitory activity of the lectin pathway, potentially providing the basis for a viable support strategy to prevent the severe complications of coronavirus infections.

## 1. Introduction

The complement system is crucial for the activation of innate and adaptive immune responses, and it plays a key role in host defense mechanisms against pathogens [1]. However, its dysregulation can lead to inflammation and its aberrant activation aggravates several lung-injury disorders [2]. Complement may be activated through the alternative, the classical and the mannose-binding lectin (MBL) pathways. Among the three pathways of complement activation, the MBL pathway is the major player to induce a proinflammatory response in viral infections [3]. Highly pathogenic human viruses such as HIV (human immunodeficiency virus), SARS-CoV (severe acquired respiratory syndrome coronavirus) and Ebola virus, all directly interact with components of the MBL pathway [1], with the dysregulation of the complement system playing main roles in the pathogenesis of respiratory disorders such as acute lung injury. Acute lung injury represents the more severe form of several viral diseases, including SARS, MERS (middle east respiratory syndrome) and COVID-19 [4,5]. Pharmacological inhibition, using known inhibitors of complement convertases C3 and C5, which are common factors to the three pathways, has already been explored as a potential therapy for SARS-CoV-2 infections with encouraging results [6].

**Figure 1:**
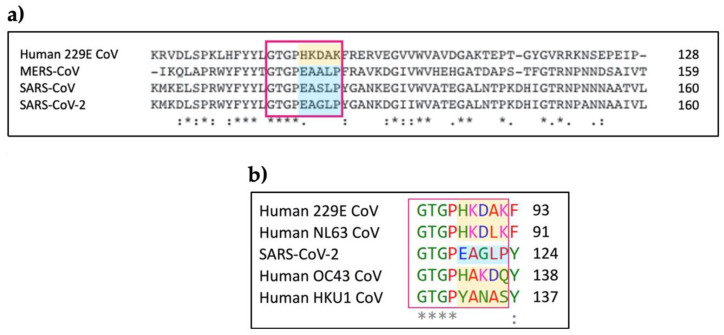
(**a**) Sequence alignment of the interacting portion of the C-terminal domain of human coronavirus N proteins. The region interacting with mannose-binding protein-associated serine protease 2 (MASP-2) is highlighted with a red box. While the amino acid composition of this region is highly conserved in highly pathogenic human coronaviruses, significant differences are found in the corresponding region of human coronaviruses associated with mild diseases, exemplified by 229E-CoV (varying portion highlighted in orange). This sequence variation has been proven to abrogate the ability to interact with MASP-2 and induce aberrant complement activation [7]. (**b**) Sequence alignment between the relevant portion of the C-terminal domain of the SARS-CoV-2 N protein and the corresponding portion of the N proteins of human coronaviruses associated with mild diseases [9]. The region interacting with MASP-2, highlighted with a red box, shows significant differences (varying portion highlighted in orange). N protein sequences were downloaded from UniProt [10]. Sequence alignments were performed with Clustal Omega [11]. In the sequence alignment, ‘*’ indicates conserved residues, ‘:’ indicates conserved substitutions, while ‘.’ indicates semi-conserved substitutions. Red indicates small and hydrophobic residues, blue indicates acidic residues, magenta indicates basic residues, green indicates residues containing hydroxyl or sulfhydryl or amine and G [11].

A conserved feature of human coronaviruses SARS-CoV, MERS-CoV and SARS-CoV-2, which is not shared by less pathogenic coronaviruses [7], is their ability to induce an excessive immune response in the host through aberrant activation of the complement system. This results in aggravated inflammation in the lungs and leads to severe pneumonia and lung injury, responsible for the high mortality rates of these viral infections [7]. Recent evidence suggests that these highly pathogenic coronaviruses trigger inflammation processes through a conserved interaction between the viral nucleocapsid (N) protein and the host MASP-2 serine protease, a key protein in the MBL pathway of complement activation. This interaction leads to excessive immune responses in the lungs, with severe consequences. This finding indicated that N protein is not only essential for the virus transcription and assembly [8] but also plays a key role in the viral pathogenesis of coronaviruses, at least partially through the interaction and the consequent exacerbated activation of MASP-2. This hypothesis was further corroborated by the observation that *MASP-2* knockout mice with induced severe pneumonia, which was boosted by SARS-CoV or MERS N protein, resulted in a higher survival rate and reduced lung damage compared to wild-type mice [7]. The viral N protein portion responsible for this interaction was recently identified, corresponding to residues 115-123 in SARS-CoV-2 (GTGPEAGLP) [7]. While the amino acid sequence for this portion is highly conserved in SARS-CoV, MERS-CoV and SARS-CoV-2, sequence alignment revealed significant variations in the corresponding sequence of the N proteins of other coronaviruses associated with mild diseases, such as human coronaviruses 229E, OC43, NL63, and HKU1 (Figure 1) [9].

**Figure 2:**
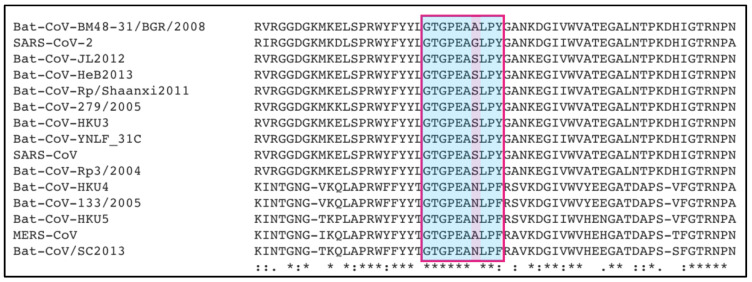
Sequence alignment between the relevant portion (a red square highlights the interacting sequence and one extra residue at the C-terminal portion, either Tyr or Phe) of the C-terminal domain of SARS-CoV-2, MERS-CoV and SARS-CoV N proteins and the N proteins of representative bat coronaviruses. The sequence conservation for the interacting portion with MASP-2 suggests the potential for these bat coronaviruses to induce severe lung injury in humans. Conserved residues are highlighted in light blue, with the only variable position highlighted in lilac. Evidence from SARS-CoV, MERS-CoV and SARS-CoV-2 indicate that variations in this position are tolerated and do not affect its binding to MASP-2. N protein sequences were downloaded from UniProt [10]. Sequence alignments were performed with Clustal Omega [11]. In the sequence alignment, ‘*’ indicates conserved residues, ‘:’ indicates conserved substitutions, while ‘.’ indicates semiconserved substitutions [11].

Remarkably, the N proteins of different bat coronaviruses not yet known to infect humans are characterized by the presence of this conserved sequence, as highlighted in Figure 2, suggesting the potential risk for emergence of new coronavirus zoonotic infections in the future, with the same serious consequences of SARS-CoV, MERS-CoV and SARS-CoV-2.

Both blocking the interaction between MASP-2 and coronavirus N proteins with anti-N or anti-MASP-2 monoclonal antibodies [7] and interfering directly with the catalytic activity of MASP-2, have been found to significantly alleviate coronavirus-induced lung injury both in vitro and in vivo [7,12], suggesting the interference with MASP-2 hyperactivation may be an effective therapeutic approach to reduce lung injury and fatality rates in coronaviral diseases.

In this study we screened in silico a drug-repurposing library of 8.736 agents that have been licensed or at least reached clinical trials, according to two parallel strategies, both aimed at identifying potential modulators of MASP-2 hyperactivation. With the first strategy, we selected potential direct inhibitors of MASP-2 at its catalytic site and evaluated them in an in vitro assay for their ability to interfere with the MBL pathway of complement activation. With the second strategy, we used molecular modelling analyses to investigate the interaction between MASP-2 and coronavirus N proteins. These studies allowed the refinement of a plausible structural model for the interaction between these two proteins, which we used to perform a second in silico screening of our drug repurposing library. Effective inhibitors of this interaction would enable the selective modulation of virus-induced immune responses, as opposed to the nonspecific effects of currently explored treatment options targeting host immune and inflammatory pathways, such as IL-6 antibodies and steroids [13,14].

## 2. Materials and Methods

### 2.1. Molecular Modelling

All molecular modelling experiments were performed on Asus WS X299 PRO Intel^®^ i9-10980XE CPU @ 3.00GHz x 36 running Ubuntu 18.04 (graphic card: GeForce RTX 2080 Ti) (Asus, Taipei, Taiwan). Molecular Operating Environment (MOE, 2019.10, Montreal, QC, Canada) [15], Maestro (Schrödinger Release 2020-2, New York, NY, USA) [16], PLANTS [17], Seesar (version 9.2, containing FlexX, Sankt Augustin, Germany) [18] and OpenEye (OEDocking 4.0.0.0, containing ScorePose) [19] were used as molecular modelling software. A virtual library of drug repurposing compounds was built in sdf (structure-data file) format by combining molecules downloaded from two different database collections containing FDA-approved drugs, clinical trial drugs, and preclinical tool compounds (DrugBank; Drug Repurposing Hub) [20,21], and then prepared using the Maestro LigPrep tool by energy minimizing the structures (OPLS_2005 force filed), generating possible ionization states at pH 7 ± 2 (Epik), generating tautomers and generating, at most, three low-energy ring conformers. All the compounds featuring chiral centers were purchased as pure enantiomers, unless otherwise indicated, in case the drug/chemical agent was used as a racemic mixture. Configurations at chiral centers were retained as per input structures.

#### 2.1.1. Docking-Based Virtual Screening on MASP-2 Catalytic Site

The virtual library of drug-repurposing compounds was screened on the catalytic site of the active form of wild-type MASP-2 (i.e., searching for direct inhibitors of MASP-2 activity) using the 1Q3X crystal structure (downloaded from the protein data bank PDB) [22]. This structure was prepared using the protein preparation tools in MOE 2019.10, and a reference ligand was added in the catalytic site using the coordinates of a co-crystallised, specific MASP-2 peptide inhibitor taken from the 3TVJ crystal structure [23]. Only the central portion of the ligand directly binding to the MASP-2 catalytic site was kept (centred on Lys30) and used to define the centre for the docking grid in Maestro. The resulting protein–ligand complex was saved in three different formats: pdb (to be used for FlexX and ScorePose rescore), mol2 (to be used for PLANTS rescore after removing the peptide ligand) and mae (to be used in Maestro to perform the docking study and XP rescoring).

The protein in the mae format was pre-processed in Maestro using the Schrödinger Protein Preparation Wizard tool, by assigning bond orders, adding hydrogens and performing a restrained energy minimisation of the added hydrogens using the OPLS_2005 force field. A 15 Å docking grid (inner-box 12 Å and outer-box 27 Å) was prepared using as the centroid the added ligand. The drug-repurposing library was docked on the active site using the Glide SP (standard precision) docking algorithm [16], keeping the default parameters, setting to five the number of output poses per input ligand to include in the solution, and performing a post-docking minimisation of each of the poses kept. The docking results obtained were then rescored using Glide XP, FlexX Score, CHEMPLP (PLANTS) and Chemgauss4 (ScorePose) scoring functions [16,17,18,19]. The values of the four different scoring functions for each docking pose were then combined together (*consensus score*) and only the docking poses falling in the top 25% of the score value range for all the four scoring functions were selected for the final visual inspection. The visual inspection process, conducted as last step, was performed using MOE 2019.10. The docking poses of the compounds obtained from the *consensus score* procedure were evaluated considering the following criteria:

- ability of a compound to overall occupy MASP-2 active site.
- number of interactions formed between the compound and the protein residues defining the site (H-bonds, pi–pi interactions, etc.).
- coverage of different chemical scaffolds, discarding similar chemical entities.

#### 2.1.2. Protein-Protein Docking between MASP-2 and SARS-CoV-2 N Protein and Druggable Site Analysis

The MASP-2 crystal structure, comprising the SP, CCP1 and CCP2 domains in complex with complement C4, was downloaded from the PDB (http://www.rcsb.org/; PDB code 4FXG [24], accessed on 30 November 2020), prepared using the MOE Protein Preparation tools, and defined as the receptor protein in the Protein-Protein Docking tool in MOE 2019.10 [15]. SARS-CoV-2 N protein crystal structure was downloaded from the PDB (PDB code 6M3M, [25]), prepared using the MOE Protein Preparation tools, and defined as the ligand protein in the MOE Protein-Protein Docking tool, specifying as the interacting portion residues 115-123 [7]. In the receptor structure 4FXG, the portions corresponding to the interacting regions between active MASP-2 and zymogen MASP-2 were excluded (residues 436–441, 621–625, 564–567, 587–591, 640–645) [22,26]. Twenty output models were generated, setting the refinement of intermediate models to none, and the refinement of final models to medium. The 20 output models were visually inspected for the predicted interactions between MASP-2 and the N protein interacting sequence (115–123), and for the presence of common interaction sites/modes recurring in multiple poses. The protein-protein docking results were combined with the prediction of potential druggable sites on the surface of MASP-2 crystal structure 4FXG, obtained with the Site Finder tool in MOE 2019.10 [15]. The predicted presence of druggable sites on the MASP-2 surface guided the selection of the most likely interaction model between the two proteins among the 20 docking outputs. In the selected model, the SARS-CoV-2 N protein was predicted to interact at the junction surface between MASP-2 domains SP and CCP2. This model was saved in mae format, preprocessed using the Schrödinger Protein Preparation Wizard by assigning bond orders, adding hydrogens and performing a restrained energy minimization of the added hydrogens using the OPLS_2005 force field, and then further optimized through molecular dynamics studies.

#### 2.1.3. Molecular Dynamics on the MASP-2-N-Protein Interaction Model

A molecular dynamics simulation was performed using the Desmond package for Molecular Dynamic (MD) simulation [16,27], employing the OPLS_2005 force field in the explicit solvent and the TIP3 water model. The initial coordinates for the MD simulation were taken from the interaction model obtained from the protein-protein docking study and the druggable site analysis. A cubic water box was used for the solvation of the system, ensuring a buffer distance of approximately 12 Å between each box side and the complex atoms. The system was neutralised adding four sodium counter-ions. The system was minimised and pre-equilibrated using the default relaxation routine implemented in Desmond. A 50 ns MD simulation was performed, during which the equation of motion was integrated using a 2 fs time step in the NPT ensemble with temperature (300 K) and pressure (1 atm) constant. All other parameters were set using the Desmond default values. Data were collected every 25 ps (energy) and every 100 ps (trajectory). The simulation was performed in triplicate, every time using a random seed as a starting point. Visualisation of the protein–protein complex and MD trajectory analyses were carried out using Maestro and MOE 2019.10. RMSD, secondary structure and protein–protein interactions analyses were performed using the Simulation Event Analysis tool and the Simulation Interaction Diagram of Desmond.

#### 2.1.4. Docking-Based Virtual Screening at the Predicted Interaction Site between MASP-2 and SARS-CoV-2 N Protein

A second in silico screening of the virtual library of drug-repurposing compounds was conducted at the MASP-2-N protein interaction interface (protein-protein interaction inhibitors) identified in the final frame of the molecular dynamic simulations described above. The interacting surface revealed on the MASP-2 protein structure was used as the receptor, while the central portion of the SARS-CoV-2 N protein interacting sequence [7], corresponding to residues 118–120, was included as the reference ligand to define the centre for the docking grid in Maestro. This complex was saved in the three different formats described above, pdb, mol2 and mae. The same docking and rescoring protocol described in Section 2.1.1. was followed.

### 2.2. Complement MBL-Pathway Inhibition Assay

Selected compounds were purchased from Generon Limited and Cambridge Biosciences, and dissolved in dimethyl sulfoxide (DMSO, Merck, Darmstadt, Germany).

The effect of selected compounds on the complement activation of the MBL pathway was evaluated by the Wieslab complement MBL pathway COMPLMP320 ELISA kit (Svar Life Sciences AB, Malmö, Skåne, Sweden), with minor modifications. The standard positive control of human normal serum provided in the kit was 1:101 diluted with the kit buffer, ensuring that only the specific MBL complement pathway was activated and incubated with 1% (*v*/*v*) of ether of the different test compounds at fixed concentrations or DMSO (the solvent of compounds) as a control. The assay was then performed according to the manufacturer’s specifications and as previously described [28]. Briefly, the test samples were added to 96-well strip plates precoated with mannan and incubated for 1 h at 37 °C. Heat-inactivated serum and only diluent buffer were also tested as negative and blank controls, respectively. Each control and compound were tested in duplicate. Coated wells were then washed with the washing buffer and incubated with a human anti-C5b-9 antibody conjugated to alkaline phosphatase (ALP) for 30 min at room temperature. After washes, the chromogenic substrate *p*-NitroPhenyl Phosphate (pNPP) was added to each well and absorbance was read at 450 nm on an ELISA plate reader (Tecan Infinite 200 PRO). In each experiment, the absorbance of the blank control was subtracted from the absorbance values of the other wells, and the activity of DMSO-treated serum samples without added inhibitor was set as 100%.

### 2.3. Statistical Analysis

Statistical analysis was carried out using GraphPad Prism 8.0 (GraphPad Software). Data are shown as the means ± standard deviations (SD). The dose-response curves and IC_50_ values of test compounds were determined by applying a nonlinear regression curve fitting (concentration of inhibitor versus normalised response with variable slope). Statistical significance was calculated by two-tailed unpaired Student’s *t*-test.

## 3. Results and Discussion

### 3.1. Docking-Based Virtual Screening of Drug-Repurposing Compounds on MASP-2 Catalytic Site

**Figure 3:**
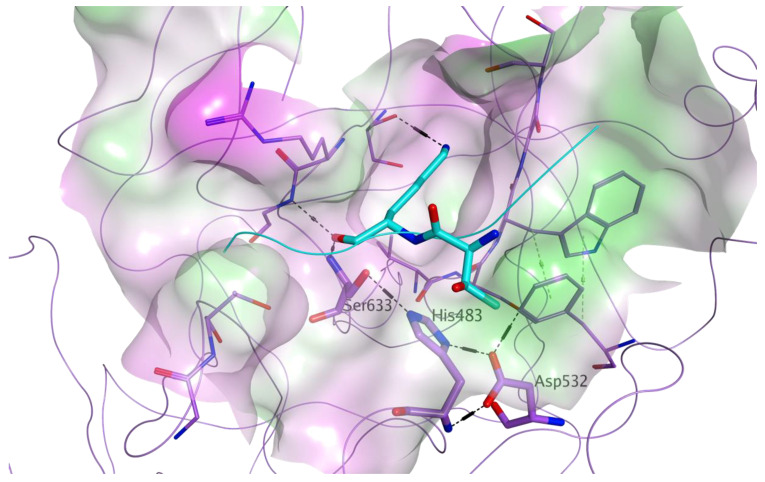
Molecular surface representation of the MASP-2 active site (PDB ID 1Q3X), with a reference known inhibitor (PDB ID 3TVJ) added to highlight the catalytic pocket. Residues of the catalytic triad (His483, Asp532, Ser633) are labelled. The surface is colour-coded according to lipophilicity (green = lipophilic, pink = hydrophilic, white = neutral). MASP-2 is represented as lilac ribbon, with carbon atoms for the amino acid residues of the active site shown in lilac. The central portion of the reference peptide inhibitor is shown as cyan ribbon, with carbon atoms for the residues shown in cyan.

A library of 8,736 commercially available drugs which have been licensed or tested in clinical trials, all small molecules available from commercial sources, was screened in silico on the catalytic site of MASP-2, in order to identify direct inhibitors of this protease. MASP-2 plays a central role in the lectin pathway of complement activation, as its proteolytic activity is responsible for the cleavage of the complement elements C2 into C2a (a function shared with MASP-1), and C4 into C4b. Once cleaved, C2a and C4b are responsible for converting the complement factor C3 into its active form C3b [23]. MASP-2 proteolytic activity is carried out by a catalytic triad formed by His483, Asp532 and Ser633. The structure of the MASP-2 active site is characterised by a high level of plasticity, which is believed to support its extreme substrate specificity [23]. For our virtual screening, the crystal structure of wild-type active MASP-2 was used (PDB ID 1Q3X) [22]. To this structure, a reference ligand was added within the active site: the known MASP-2 peptide inhibitor, SGMI-2, whose coordinates were taken from the 3TVJ crystal structure [23]. This is a substrate-like, monospecific MASP-2 inhibitor, which was developed to elucidate the specific roles of MASP-2 in the lectin-pathway of complement activation [23]. Figure 3 shows the active site of MASP-2 in the 1Q3X structure, with the central portion of the reference ligand added.

**Figure 4:**
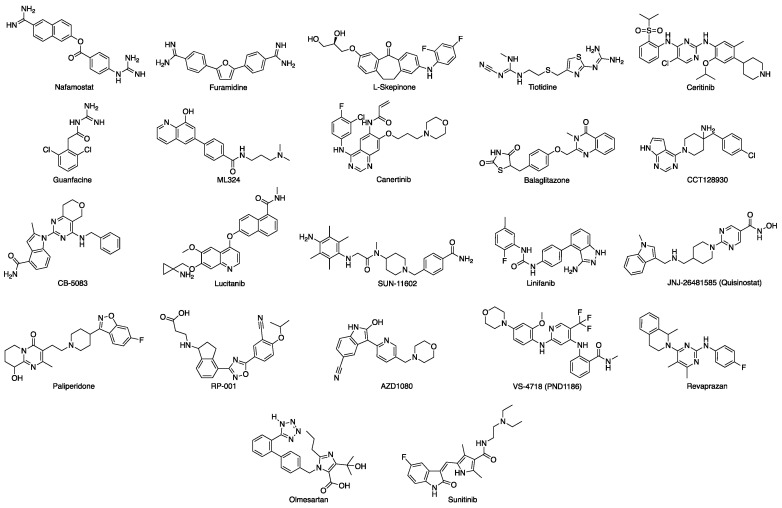
Chemical structures of the drug repurposing compounds chosen for the catalytic site of MASP-2.

The Glide Standard Precision docking tool (SP) [16], which uses the Glide-SP scoring function, was employed to virtually screen the drug-repurposing database according to a molecular docking simulation. In order to avoid any potential bias associated with the use of a single docking program/scoring function, all docking results (docking poses) obtained were then rescored using four different scoring functions: Glide XP [16], CHEMPLP (PLANTS) [17], FlexX Score (Seesar) [18] and Chemgauss4 (OpenEye ScorePose) [19]. After applying a *consensus score* procedure (Materials and Methods 2.1.3), 1,314 molecules were chosen. These compounds were visually inspected to assess their overall predicted occupation of the MASP-2 active site, and to consider their potential interactions with the amino acid residues defining this binding pocket. Following this evaluation, twenty-two virtual hits were selected as potential MASP-2 inhibitors and purchased (Figure 4).

**Figure 5:**
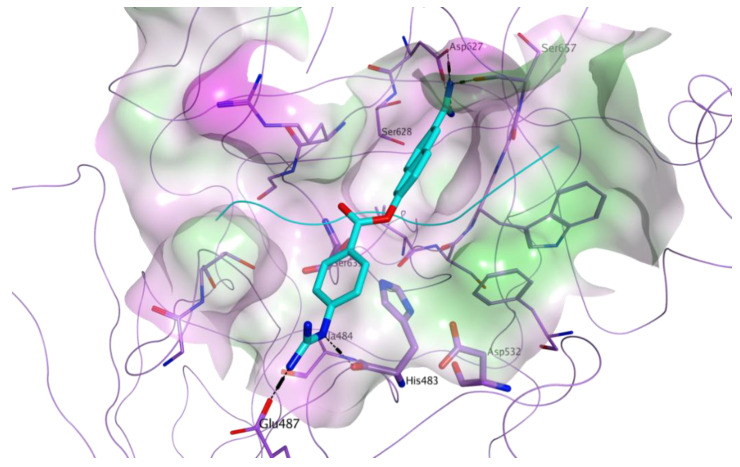
Predicted binding of nafamostat (carbon atoms in cyan) to the active site of MASP-2. The catalytic site is represented as molecular surface, colour-coded according to lipophilicity (green = lipophilic, pink = hydrophilic, white = neutral). MASP-2 is represented as lilac ribbon, with carbon atoms for the residues shown in lilac. The reference peptide inhibitor is shown as cyan ribbon.

All the selected compounds showed an optimal occupation of the MASP-2 catalytic site, with the potential of making direct interactions with different residues within the pocket. The predicted binding mode for a representative compound, nafamostat, is shown in Figure 5. This molecule shows a good fitting of the target pocket, with the potential of making multiple direct hydrogen bonds and electrostatic interactions through its two guanidine terminal groups, with the backbone carbonyl groups of the catalytic histidine (His483), with the side chain hydroxyl group of Ser657 and with the side chain carboxylate groups of Glu487 and Asp627. Rescoring values for all final choices are listed in the Supporting Information (Table S1).

Among the 22 compounds selected and purchased for this site, nafamostat is a known serine protease inhibitor, approved for the clinical use as an anticoagulant and to treat acute pancreatitis, which has recently been shown to have inhibitory activity on the complement system, with a positive effect for elderly patients with COVID-19-associated pneumonia [29]. This was the best-performing compound for MASP-2 active site in our computational study. Therefore it was kept in the final selection, also envisaging the possibility to consider it as a potential validation tool for the use of our assay to screen for inhibitors of the MBL pathway of complement activation. Nafamostat has also been found to block the fusion step of viral entry of SARS-CoV-2 in in vitro studies by inhibiting the protease activity of the host transmembrane protease serine 2 (TMPRSS2) [30]. In addition, L-skepinone has been reported to display anti-SARS-CoV-2 activity in a cell-based assay [31], while sunitinib has been found to reduce infections by SARS-CoV, MERS-CoV and SARS-CoV-2, partially by inhibiting the phosphorylation of AP2M1 [32].

### 3.2. Identification of Inhibitors of the Complement MBL Pathway

**Figure 6:**
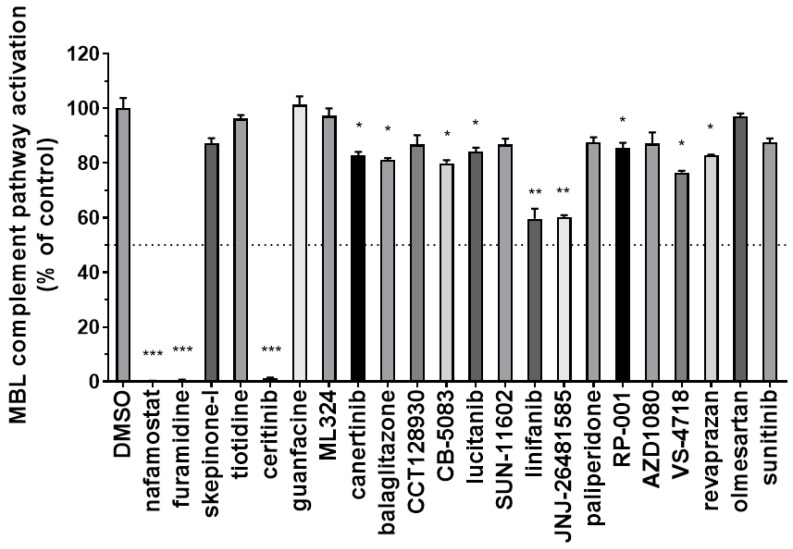
Inhibitory effect of the 22 selected compounds on the activation of the MBL complement pathway. Test compounds were dissolved in pooled normal human serum at 100µM and tested in the Wieslab ELISA-based kit to assess their potential to inhibit the complement activation of the MBL pathway. The MBL pathway activation detected by anti-C5b-9 ALP antibody in samples treated with DMSO was set as 100%. All data shown represent the means ± SD of data tested in duplicate. Data from each compound was analyzed by a Student’s t-test versus that of DMSO-treated control. * *p* < 0.05; ** *p* < 0.01; *** *p* < 0.001.

Since MASP-2 plays a critical role in the initiation of complement activation through the MBL pathway, we tested the ability of the 22 drug-repurposing compounds to interfere with this pathway in the Wieslab MBL complement kit. Mannan-precoated wells were incubated with pooled normal human serum containing the test compounds at a fixed concentration of 100 µM or DMSO as a control (Figure S1). The inhibition of the MBL pathway activation was then detected with an alkaline phosphatase anti-C5b-9 human antibody. Results obtained at this concentration are summarised in Figure 6.

**Figure 7:**
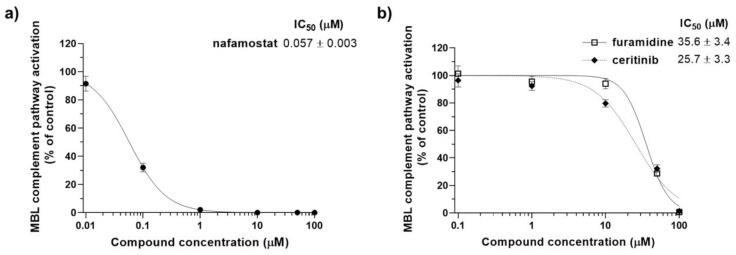
Dose-dependent inhibition of the MBL complement activation by the test compounds. Different concentrations of nafamostat (**a**) along with furamidine and ceritinib (**b**) were added in normal human serum and tested in the Wieslab ELISA kit for the MBL complement pathway activation. The IC_50_ values, corresponding to the compound concentrations that inhibit by 50% the MBL pathway activation, are reported for all the three compounds. Data shown represent the means ± SD of the experiments performed in duplicate.

In this primary screening, 12 of the test compounds showed at least partial and significant inhibition of the MBL pathway. The most potent compounds identified in this screening were nafamostat, as partially expected, furamidine, and ceritinib, which completely abolished the complement activation of the MBL pathway at 100 µM. In addition, also linifanib and JNJ-26481585 (quisinostat) showed the potential to interfere with this pathway, as they both reached almost 50% inhibition at the tested concentration. While we aim to further explore the inhibitory effect of linifanib and quisinostat in different assays, in the first instance we selected the three most potent hits, nafamostat, furamidine and ceritinib, to perform a dose-response analysis and calculate IC_50_ values in the same assay system. Figure 7 summarises the results obtained for these three hits tested at different concentrations again in the Wieslab MBL complement ELISA.

In this further evaluation, nafamostat emerged as the most potent compound, with an IC_50_ value of 0.057 µM (Figure 7a). Although its potential to inhibit the activation of the complement pathway was expected, as nafamostat acts as a serine protease inhibitor and is known to attenuate complement activation [33], the effect observed in our assay may already represent a validation of this protocol for screening and identifying potential inhibitors of the complement MBL pathway. In addition, as nafamostat exhibited clinical efficacy in COVID-19 patients [29] and showed to inhibit the replication of SARS-CoV-2 in vitro studies with a very high selectivity index (i.e., >11,000) [34], the results obtained in this assay have the potential to provide a preliminary proof of concept for our approach. Finally, we suggest that the complement inhibitory activity previously found for nafamostat may be related, at least in part, to a direct inhibition of the MASP-2 catalytic effect. Further investigations are currently ongoing in our research group to assess the robustness of this hypothesis and will be published in due course. Additional in vivo experiments are also needed to further confirm the therapeutic potential, and safety, of the proposed approach.

**Figure 8:**
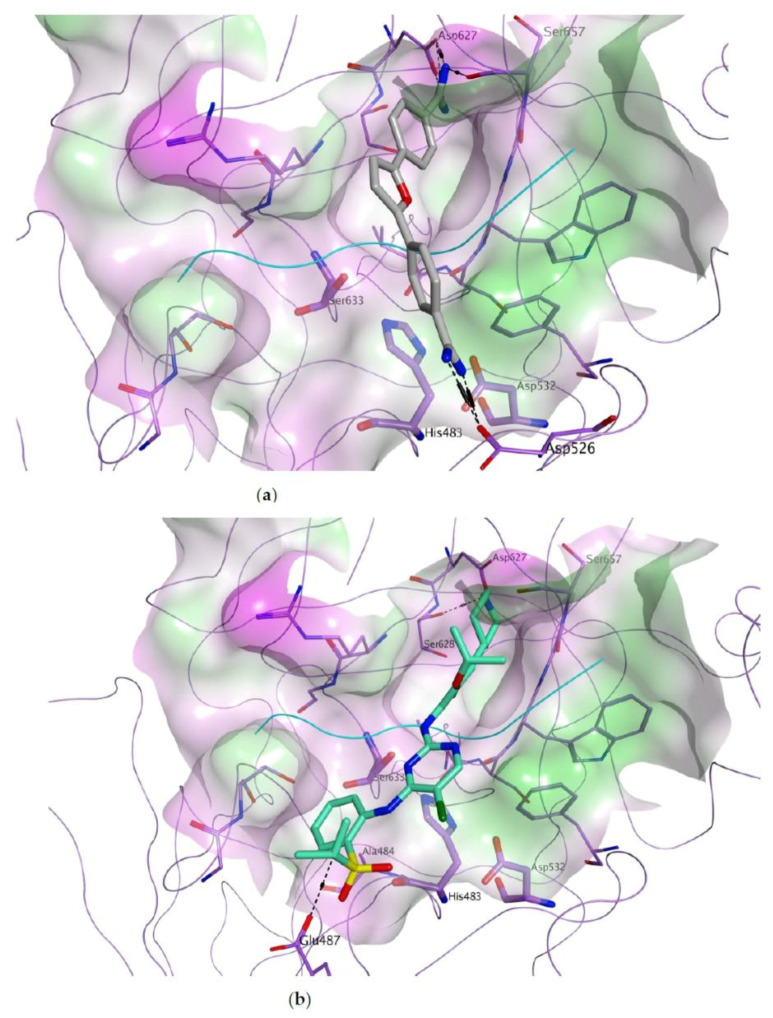
Predicted binding of furamidine ((**a**), carbon atoms in grey) and ceritinib ((**b**), carbon atoms in green) to the active site of MASP-2. The catalytic site is represented as molecular surface, colour-coded according to lipophilicity (green = lipophilic, pink = hydrophilic, white = neutral). MASP-2 is represented as lilac ribbon, with carbon atoms for the residues shown in lilac. The reference peptide inhibitor is shown as cyan ribbon.

Although less potent than nafamostat, furamidine and ceritinib also specifically inhibit the MBL pathway of complement activation in a dose-dependent manner, both displaying IC_50_ values in the micromolar range (Figure 7b). To the best of our knowledge, their inhibitory effects on the complement activation cascade have not been previously reported. Their predicted binding to MASP-2 catalytic site is shown in Figure 8.

Both furamidine and canertinib are associated with an optimal fitting of the MASP-2 active site. Furamidine (Figure 8a) has the potential to engage in multiple electrostatic and H-bond interactions through its guanidine groups with the side chain carboxylate groups of Asp526 and Asp627, and with the backbone carbonyl group of Ser657. Ceritinib (Figure 8b) is predicted to form a hydrogen bond with the side chain hydroxyl group of Ser628, a hydrophobic interaction with the side chain of Ala484 and a hydrogen bond with the side chain of Glu487, through the acidic proton of its sulfo-isopropyl group. In the case of these two hit molecules, further investigations are currently ongoing to assess their specific inhibition of MASP-2 catalytic activity, and their potential effects on related serine-proteases, including both the complement system and the coagulation cascade.

### 3.3. Molecular Model for the Protein-Protein Interaction between MASP-2 and Coronaviral N-Proteins

As a parallel strategy to the identification of direct inhibitors of MASP-2 catalytic activity, we carried out a series of molecular modelling simulations to elucidate the interaction between MASP-2 and coronavirus N-proteins, and to suggest potential inhibitors of this interaction within our drug-repurposing library of compounds.

#### 3.3.1. Protein-Protein Docking between MASP-2 and SARS-CoV-2 N Protein

**Figure 9:**
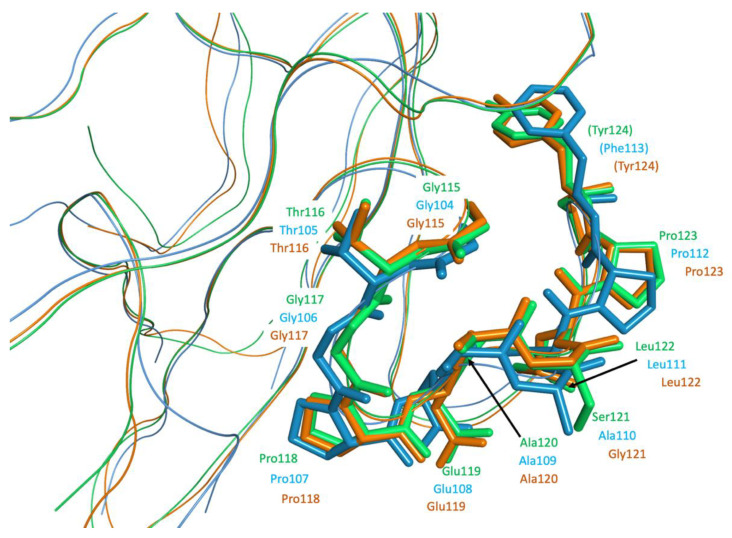
Ribbon representation of the superimposed N protein structures of SARS-CoV (PDB ID 2OFZ, ribbon and atoms in green) [35], MERS-CoV (PDB ID 6KL2, ribbon and atoms in light blue) [36], and SARS-CoV-2 (PDB ID 6M3M, ribbon and carbon in orange) [25]. Residues of the conserved, exposed region for binding to MASP-2 are shown as bold sticks and labelled for the corresponding viruses.

The portion responsible for the interaction with MASP-2 has recently been defined within the structure of coronavirus N proteins [7]. It corresponds to residues 115-123 in SARS-CoV and SARS-CoV-2 N proteins, and to residues 105-112 in MERS-CoV N protein. Figure 9 shows the structural superimposition of these three N proteins, highlighting the overall preservation of the three-dimensional features of the interacting sequence within the three viruses.

The corresponding portion of MASP-2 interacting with coronaviral N proteins has been narrowed to the CCP1-CCP2-SP C-terminal region [7], but the specific interacting residues have yet to be identified. In order to gain insights on the binding between MASP-2 and coronaviral N proteins, a protein-protein docking study was carried out in MOE 2019.10 [15], using SARS-CoV-2 N protein (PDB ID 6M3M) as a model protein for the three highly pathogenic coronaviruses. For this study, the crystal structure of the CCP1-CCP2-SP portion of the active form of MASP-2 in complex with one of its substrates, C4, was used (PDB code 4FXG) [24]. The presence of the substrate C4 was preserved during the docking analysis to exclude from the docking results any possible binding mode involving the MASP-2 region where the substrate binds. In addition, the regions of MASP-2 where the binding between zymogen form and active form of MASP-2 occurs to promote MASP-2 autoactivation, were also excluded from our docking study, as its interaction with coronavirus N proteins significantly enhances MASP-2 autoactivation [7]. Finally, as the CUB1-EGF-CUB2 region of MASP-2 is linked to the N-terminal portion of the CCP1 domain, the terminal part of this subregion was also excluded from our study. From the protein-protein docking simulation, a database of 20 final models for the interaction between the two proteins was obtained (Supporting Information, Table S2). All models were visually inspected to evaluate potential interactions between the SARS-CoV-2 N protein interacting sequence (residues 115–123) and MASP-2, and for the presence of common binding regions on MASP-2 recurring in multiple models, i.e., common binding modes repeated in different docking poses. Interestingly, a common binding area, located at the interface between MASP-2 domains CCP2 and SP, was shared by multiple models.

**Figure 10:**
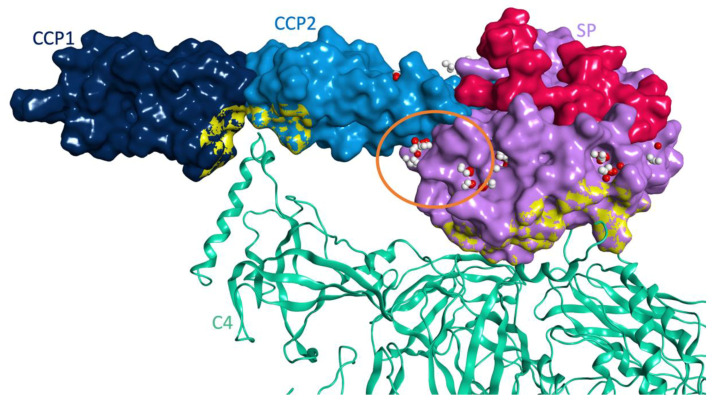
Surface representation of MASP-2 C-terminal region in complex with the substrate C4 (PDB code 4FXG). MASP-2 domains are represented as molecular surface: CCP1 in dark blue, CCP2 in light blue and SP in lilac. C4 is represented as green ribbon. The regions excluded from the docking study with SARS-CoV-2 N protein are highlighted as an overlapping yellow surface (MASP-2 contact surface with C4), or as a pink surface (activation peptide and interacting portions between the two forms, zymogen and active enzyme, of MASP-2). Druggable sites located on the MASP-2 C-terminal region available for binding to coronaviral N proteins are shown (PDB code 4FXG), as identified using the Site Finder tool in MOE 2019.10 [15]: white spheres represent zones suitable for hydrophobic interactions while red spheres represent suitable centers for the potential formation of hydrogen bonds or electrostatic interactions.

Next, we analyzed the surface of MASP-2 in the 4FXG crystal structure with the Site Finder tool in MOE 2019.10 [15] to identify possible druggable sites within the region of space available for binding to coronaviral N proteins. Figure 10 shows the structural domains of MASP-2 in the 4FXG crystal structure, the corresponding portions excluded from the docking simulation and all the potential druggable sites detected with the Site Finder analysis.

**Figure 11:**
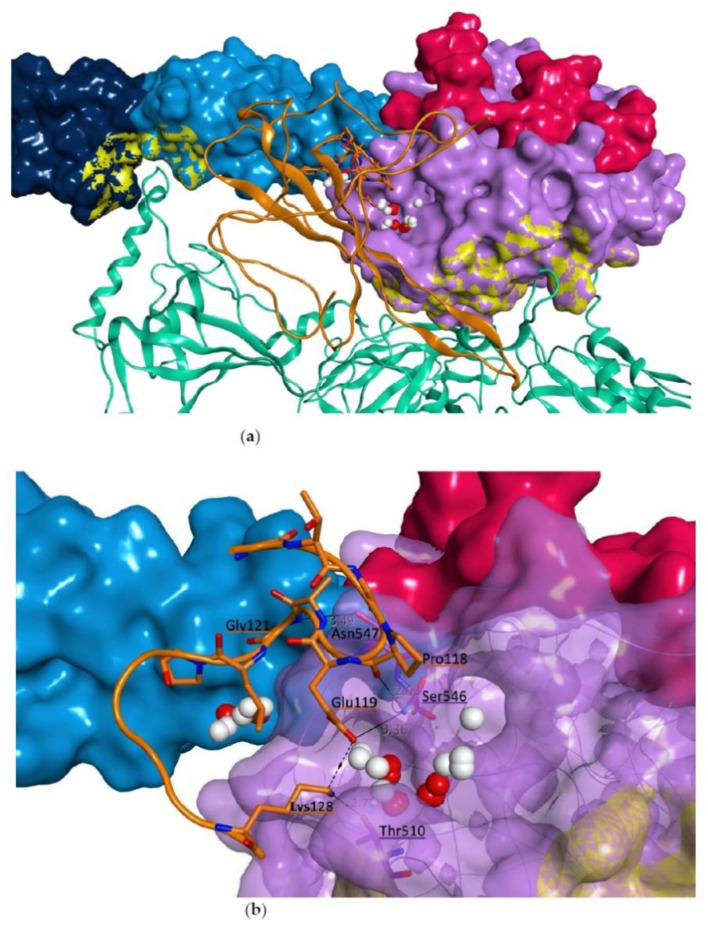
Protein-protein docking model between MASP-2 and SARS-CoV-2 N protein (orange ribbon) chosen for structural optimization by molecular dynamics: (**a**) overall overview of the binding mode, and (**b**) zoom on the predicted interaction site of the N protein interacting sequence (only residues 115-123 are shown, with carbon atoms in orange and orange ribbon, along with Lys128). MASP-2 domains are represented as molecular surface: CCP1 in dark blue, CCP2 in light blue and SP in lilac. C4 is represented as green ribbon. The druggable site at the interacting surface with the N protein is represented as white (hydrophobic interactions) and red (polar interactions) spheres.

Interestingly, one main druggable site, highlighted with an orange circle in Figure 10, is located at the interface between the CCP2 and SP domains in proximity to where the SARS-CoV-2 N protein is predicted to bind in the recurring binding mode from the protein-protein docking simulation. In this binding pose, the N protein interacting sequence (residues 115-123) is placed in close proximity to this main druggable site, suggesting its likelihood in predicting a realistic interaction model between the two proteins. This model was used for further structural optimization by molecular dynamics first, and then to perform the virtual screening analysis of our drug-repurposing virtual library, to search for potential inhibitors of this protein-protein interaction. Figure 11 shows a zoom of the predicted interaction surface between MASP-2 and SARS-CoV-2 N protein in our docking model. On top of occupying the druggable site between the CCP2 and SP domains, four direct hydrogen bonds between the two proteins are present: Asn547 (SP) directly interacts with Pro118 and Glu119, Ser546 (SP) makes a H-bond with the backbone of Gly121, while Thr510 (SP) is H-bonded to Lys128, which is close in space to the known interacting sequence residues 115–123.

#### 3.3.2. Molecular Dynamics Studies for Structural Optimization of the Interaction Model between MASP-2 and SARS-CoV-2 N Protein

In order to optimize and validate the interaction model between the two proteins, a 50 ns molecular dynamics simulation was performed on this complex using the Desmond software package [16,27]. This simulation was run in triplicate. The system consistently reached stabilization after an initial 10 ns of equilibration with the simulation converging around a fixed RMSD value, as shown by a small C-alpha RMSD variation for both proteins analysed, MASP-2 and SARS-CoV-2 N protein (Figure S2). The geometry of the druggable site on the surface of MASP-2, and the presence and nature of direct interactions between the two proteins, mainly focusing on those involving the N protein residues 115-123, were monitored throughout the simulation. During the MD analyses, the N protein interacting sequence tended to optimize its occupation of the druggable site, maintaining a stable position, and to adjust its relative orientation to maximize contacts with the surrounding MASP-2 residues. The presence of SARS-CoV-2 N protein also induced a structural and spatial optimization of the MASP-2 residues defining the druggable site, which became more definite towards the end of all three simulations.

Distinct direct contacts between the N protein interacting sequence and residues of MASP-2, mainly hydrogen bonds, were consistently present during the entire MD simulation, providing useful insights for searching for inhibitors. In particular, following analysis of the simulations with the Simulation Interaction Diagram function of Desmond [27], the MASP-2 residues Arg376, Tyr401, His508, Asn545, Ser546 and Asn547 were involved in hydrogen bonds with the interacting sequence of SARS-CoV-2 N protein for a relevant fraction of the simulation time (Supporting Information, Figure S3). In addition, MASP-2 residues involved in likely hydrophobic interactions with the N protein interacting sequence were also revealed: Phe400, Tyr401 and Val543 (Supporting Information, Figure S3). Representative individual frames of the triplicate simulation were systematically visually inspected for the presence of additional contacts between MASP-2 and the N protein interacting sequence using MOE 2019.10 [15]. Findings from this visual inspection are reported in the Supporting Information (Table S3).

**Figure 12:**
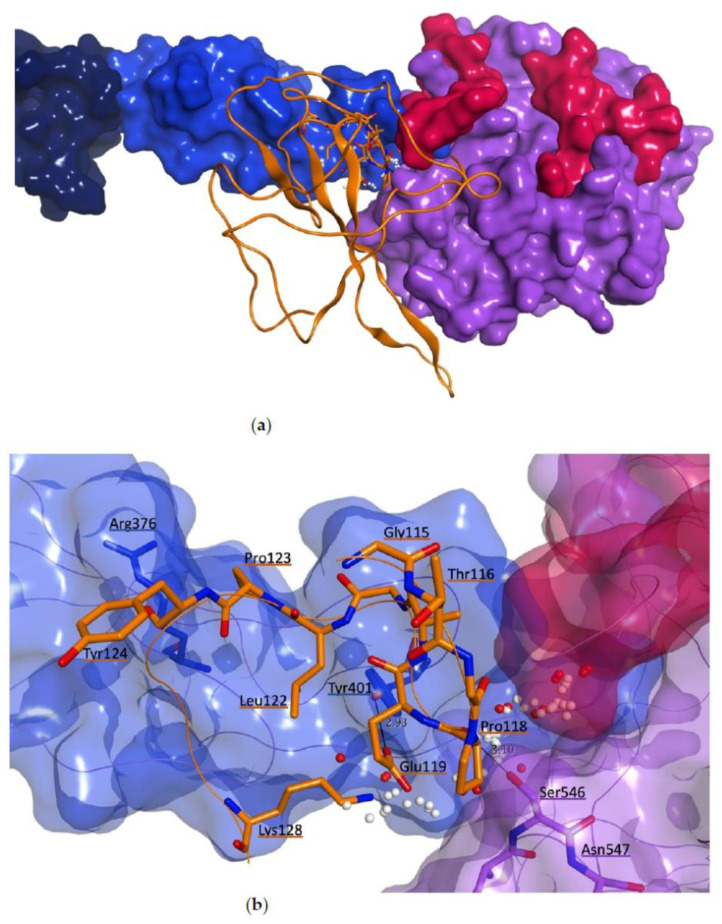
Predicted binding mode between MASP-2 C-terminal region and SARS-CoV-2 N protein (orange ribbon) after MD simulation: overview of the final interacting complex (**a**) and zoom on the interaction site defined by the interacting sequence of SARS-CoV-2 N-protein (**b**). MASP-2 domains are represented as molecular surface: CCP1 in dark blue, CCP2 in light blue and SP in lilac. The druggable site at the interacting surface with the N protein interacting sequence is represented as white (hydrophobic interactions) and red (polar interactions) spheres. Carbon atoms of the N protein residues are in orange.

The final optimized complex after molecular dynamics, with a zoom on the druggable site located at the interaction surface between MASP-2 and the N protein interacting sequence, is shown in Figure 12.

#### 3.3.3. Virtual Screening on the Predicted Interaction Site between MASP-2 and SARS-CoV-2 N Protein

**Figure 13:**
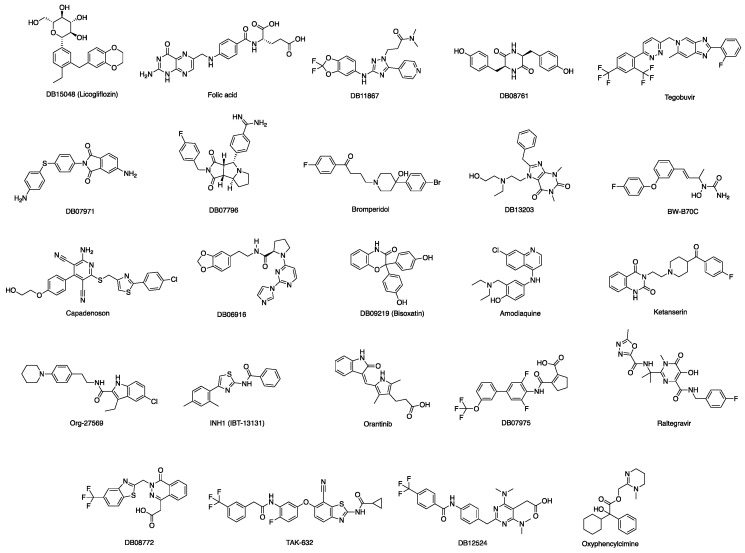
Chemical structures of the drug repurposing compounds chosen for the predicted interaction site between MASP-2 and SARS-CoV-2 N protein.

A drug-repurposing library of 8736 licensed drugs and clinical agents was analyzed according to a second virtual screening with the aim of identifying small molecules able to bind at the interaction site defined by the SARS-CoV-2 N protein interacting sequence on the MASP-2 surface in the final, optimized complex model after MD. Following the same approach described above for the MASP-2 active site (3.1), after *consensus scoring* a total of 851 molecules were chosen as potential disruptors of the interaction between SARS-CoV-2 N protein and MASP-2. These compounds were visually inspected for their overall occupation of the target druggable site on MASP-2 where the N-protein interacting sequence is predicted to bind. After visual inspection, twenty-four virtual hits were selected and purchased (Figure 13).

**Figure 14:**
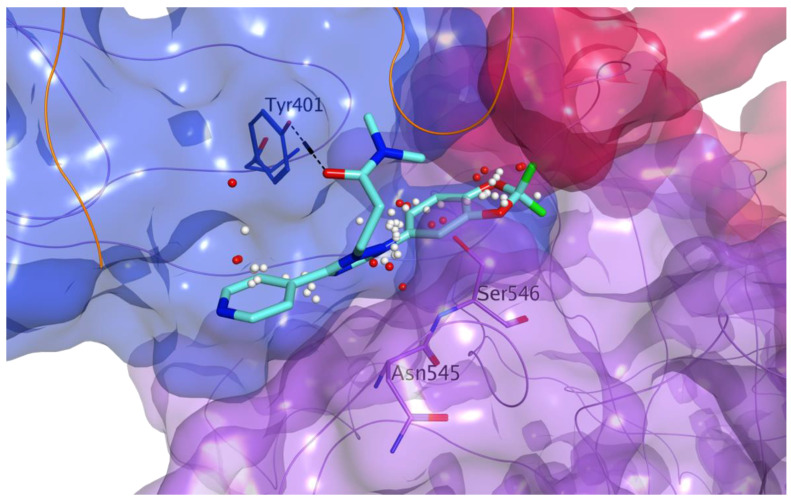
Predicted binding of DB11867 (carbon atoms in cyan) to the druggable site at the predicted interaction surface between MASP-2 and SARS-CoV-2 N protein. MASP-2 domains are represented as molecular surface: CCP2 in light blue (carbon atoms for residues shown in light blue) and SP in lilac (carbon atoms for residues shown in lilac). The druggable site is represented as white (hydrophobic interactions) and red (polar interactions) spheres. SARS-CoV-2 N protein is represented as orange ribbon.

All these molecules showed an optimal overall occupation of the druggable site in our model, with the potential of forming hydrogen bonds and hydrophobic interactions with different residues of MASP-2, as exemplified in Figure 14, by the predicted binding of DB11867. On top of showing a good fitting of the site, this compound is also predicted to make a direct hydrogen bond with the side chain hydroxyl group of Tyr401 (CCP2). By interacting with this portion of MASP-2, these compounds could potentially prevent coronavirus N proteins from binding, thus interfering specifically with the virus-induced hyperactivation of MASP-2. Rescoring values for all final choices are listed in the Supporting Information (Table S4).

Among the 24 drug-repurposing molecules selected, some agents have already been proposed as candidates for COVID-19 repurposing. Assumption of folic acid has been observed to protect pregnant women from SARS-CoV-2 infection, with contrasting evidence suggesting that it may either inhibit the furin protease, needed by the virus to enter the host cell, or the protease 3CL^pro^, essential for viral replication [37]. As a small-molecule antiviral agent, tegobuvir has been evaluated in clinical trials for COVID-19 [38], while bisoxatin (DB09219) has been suggested as a potential inhibitor of SARS-CoV-2 spike protein following a computational approach [39]. Amodiaquine has been found to inhibit SARS-CoV-2 replication in vitro [40], ketanserin has been suggested as a potential additive drug to improve the ventilation/perfusion mismatch in patients with COVID-19 [41] and, finally, the anti-HIV drug raltegravir has been proposed as a potential 3CL^pro^ inhibitor following a computational approach [42].

We are currently working towards the development of an ELISA assay to first confirm the interaction between the MASP-2 catalytic domain and SARS-CoV-2 N protein, and then to evaluate the twenty-four selected candidates for their inhibition of this interaction.

## 4. Conclusions

MASP-2 represents an important target for the development of therapeutic measures to interfere with the severe consequences of infections with highly pathogenic coronaviruses SARS-CoV, MERS-CoV and SARS-CoV-2. By directly interacting with MASP-2 through their N proteins, these viruses induce MASP-2 hyperactivation, leading to severe lung injury in patients. In this work, we followed two parallel strategies to interfere with the MASP-2 hyperactivation induced by these viruses. First, we performed a docking-based virtual screening of a virtual library of 8,736 licensed drugs and clinical agents on the catalytic site of MASP-2, searching for direct inhibitors of its proteolytic activity. For this in silico study, we selected and purchased a total of 22 drug-repurposing candidates. These molecules were preliminarily evaluated in an in vitro assay to determine their potential interference with the MBL pathway of complement activation: different candidates were found to inhibit this pathway. In particular, we observed a potent effect for nafamostat, which had been at least partially described previously, and an interesting effect for furamidine and ceritinib, which both displayed IC_50_ values in the micromolar range. Complement inhibitory activity for these two licensed drugs has not been described previously. Currently, we are evaluating all our chosen candidates in further assay systems for their direct and selective inhibition of MASP-2 catalytic activity. However, it should be noted that ceritinib has shown to clinically induce interstitial lung disease, limiting its potential use against COVID-19 [43]. In parallel, we also performed a series of protein-protein docking studies and molecular dynamics simulations, to investigate the interaction between coronavirus N proteins and MASP-2, and to propose this interaction as a target for the identification of specific anti-coronavirus agents. While the amino acid sequence responsible for the interaction has been identified for coronaviral N proteins, information on the interacting portion of MASP-2 has yet to be reported. Nonetheless, the MASP-2 interacting portion has been previously narrowed down to the CCP1, CCP2 and SP domains. In this work, we revealed a plausible interaction site on the surface of MASP-2, where the interacting sequence of coronavirus N proteins is likely to bind. Our model provides a useful tool for the identification or the design of specific inhibitors of this interaction for therapeutic purposes. With the aim to identify drug-repurposing candidates able to prevent this protein-protein interaction, we used our model to perform a second, independent virtual screening of the drug-repurposing library. We identified 24 promising candidates as potential inhibitors of this interaction, and are currently setting up multiple assays for their biochemical function evaluation. Overall, we highlight here the potential of our approach to pave the way for the development of a support therapeutic measure for present and future-emerging coronaviral infections, to decrease the severe consequences and high mortality rates of these viral diseases. The potent activity observed for nafamostat in our preliminary assay provides an initial proof of concept for our strategy. In addition, our promising findings for nafamostat, furamidine and ceritinib also suggest the potential of successfully finding additional, novel therapeutic agents by extending our *in silico* methodology to the evaluation of much bigger libraries of drug-like compounds. Finally, these initial data represent an encouraging confirmation of how computer-aided methods can guide the identification of biologically active compounds, further supporting the likelihood of identifying effective, specific protein-protein interaction inhibitors of MASP-2 interaction with coronavirus N proteins.

## Data Availability

The data presented in this study are available on request from the corresponding author.

## Supplementary Materials

The following are available online at https://www.mdpi.com/1999-4915/13/2/312/s1, Table S1: Rescoring values for drug-repurposing compounds chosen for MASP-2 active site, Table S2: Protein-protein docking results (models) between MASP-2 and SARS-CoV-2 N protein, Figure S1: Effect of serum heat-inactivation and DMSO on the MBL complement pathway activity, Figure S2: RMSD values for the alpha-carbon atoms of the two proteins during MD simulations, Figure S3: Direct contacts between MASP-2 and the interacting sequence of SARS-CoV-2 N protein during the MD simulation, Table S3: Direct contacts between MASP-2 and SARS-CoV-2 N proteins in discrete MD frames, and MASP-2 residues defining the interaction site between the two proteins, Table S4: Rescoring values for drug-repurposing compounds chosen for the interaction site between MASP-2 and SARS-CoV-2 N protein.
