# Supplementary material for: Targeting the Complement Serine Protease MASP-2 as a Therapeutic Strategy for Coronavirus Infections
Source: Viruses. 2021 Feb 17;13(2):312. doi: 10.3390/v13020312 (PMC7923061; doi:10.3390/v13020312)
Supplement: Supplementary file 1 [file viruses-13-00312-s001.pdf]

Targeting the complement serine-protease MASP-2 as a therapeutic strategy for coronavirus diseases

Ben M. Flude[1], Giulio Nannetti[2], Paige Mitchell[1], Nina Compton[3], Chloe Richards[4], Meike Heurich[2], Andrea Brancale[2,*], Salvatore Ferla[5], and Marcella Bassetto[1]

[1] Department of Chemistry, College of Science and Engineering, Swansea University, Swansea, UK
[2] School of Pharmacy and Pharmaceutical Sciences, Cardiff University, Cardiff, UK
[3] Coleg Gwent, Crosskeys Campus, Crosskeys, UK
[4] Ysgol Gyfun Rhydywaun, Rhondda Cynon Taf, UK
[5] Swansea University Medical School, Swansea, UK

* Correspondence: brancalea@cardiff.ac.uk

Supporting Information

Contents

Table S1: Rescoring values for drug-repurposing compounds chosen for MASP-2 active site.

Table S2: Protein-protein docking results (models) between MASP-2 and SARS-CoV-2 N protein.

Figure S1: Effect of serum heat-inactivation and DMSO on the MBL complement pathway activity

Figure S1: RMSD values for the alpha-carbon atoms of the two proteins during MD simulations.

Figure S2: Direct contacts between MASP-2 and the interacting sequence of SARS-CoV-2 N protein during the MD simulation.

Table S3: Direct contacts between MASP-2 and SARS-CoV-2 N proteins in discrete MD frames, and MASP-2 residues defining the interaction site between the two proteins.

Table S4: Rescoring values for drug-repurposing compounds chosen for the interaction site between MASP-2 and SARS-CoV-2 N protein.

Table S1

Rescoring values for the drug repurposing final choices for the catalytic site of MASP-2.

Compound	Rescoring values, kcal/mol			
	Glide XP	PLANTS	FlexX	ScorePose
Nafamostat	-8.4292631	-77.382202	-29.23	-14.948345
Furamidine	-7.6509728	-81.5009	-33.458	-10.458287
Skepinone-1	-7.4871306	-84.484703	-17.99	-11.943799
Tiotidine	-5.7613311	-74.677696	-29.006001	-11.650853
Ceritinib	-7.4828296	-85.0252	-23.82	-8.6786385
Guanfacine	-8.0983143	-69.356201	-19.563	-11.27206
ML324	-4.2543254	-82.982399	-22.639	-12.195214
Canertinib	-4.5961032	-85.014503	-21.056	-10.462101
Balaglitazone	-5.2521544	-79.750099	-19.941	-10.705989
CCT128930	-7.3585343	-71.544601	-19.858999	-9.128973
CB-5083	-4.5376992	-75.287498	-22.572001	-10.545598
Lucitanib	-7.5688577	-78.883698	-15.091	-8.9847565
SUN-11602	-5.1951108	-82.136597	-20.254	-8.5957499
Linifanib	-4.4032121	-72.777	-23.044001	-9.319706
JNJ-26481585	-5.3877602	-72.921402	-20.924999	-8.0121317
Paliperidone	-5.4913545	-73.477898	-15.971	-8.5497789
RP-001	-4.1750298	-76.439499	-17.313	-8.7906342
AZD1080	-4.2881861	-75.641899	-17.223	-8.4997702
VS-4718	-5.019186	-74.370102	-17.351	-7.714396
Revaprazan	-4.8230472	-70.754402	-16.761999	-8.5200548
Olmesartan	-4.8132172	-70.759399	-13.546	-8.795146
Sunitinib	-6.0446553	-74.079102	-19.391001	-12.049711

Table S2

Protein-protein docking results between MASP-2 (PDB ID 4FXG) and SARS-CoV-2 N protein (6M3M), obtained with MOE 2019.10.

Complex	Structure*	MOE Docking Score	Complex	Structure*	MOE Docking Score
1	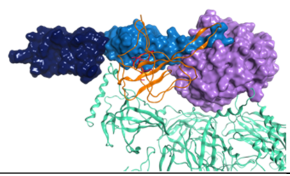	-78.438843	11	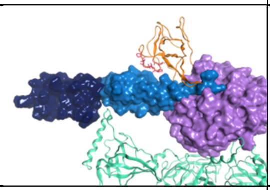	-48.151939
2	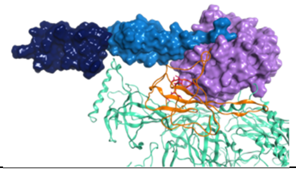	-64.966309	12	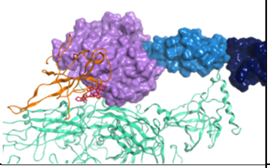	-47.884235
3	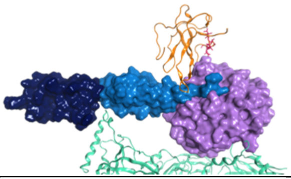	-56.666553	13	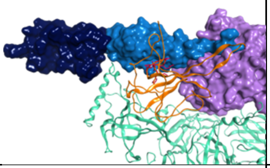	-46.32021
4	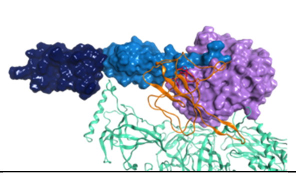	-56.060783	14	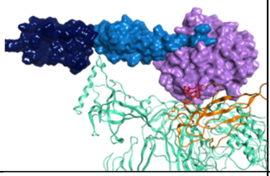	-44.49239
5	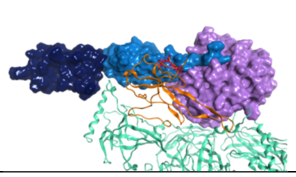	-55.253914	15	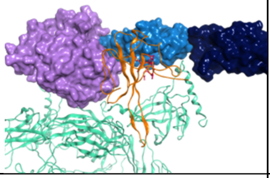	-43.264782
6	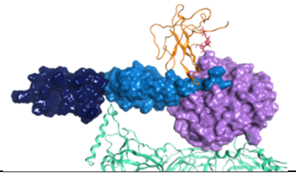	-53.31538	16	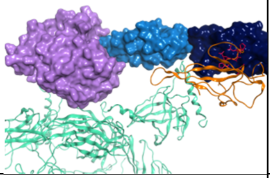	-41.950214
7	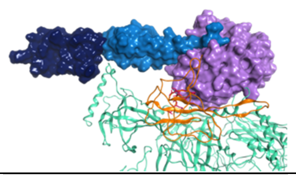	-51.986874	17	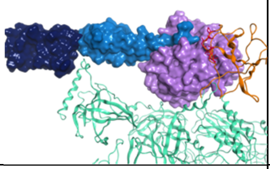	-40.230049
8	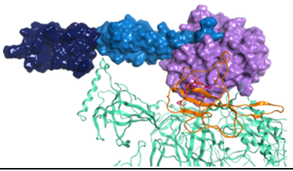	-49.597775	18	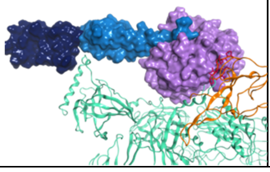	-35.882229

9	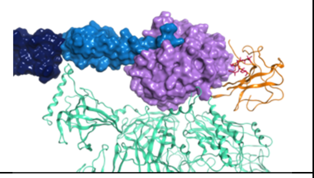	-48.835548	19	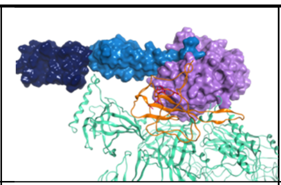	-30.870058
10	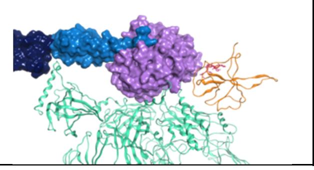	-48.414017	20	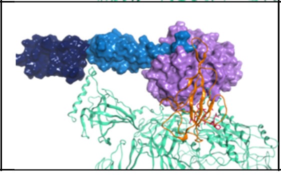	-23.445593

* MASP-2 domains are represented as molecular surface: CCP1 in dark blue, CCP2 in light blue and SP in lilac. C4 is represented as green ribbon. SARS-CoV-2 N protein is represented as orange ribbon, with atoms if the interacting sequence (res. 115-123) shown in pink.

Figure S1

Effect of serum heat-inactivation and DMSO on the MBL complement pathway activity. Positive standard non-heat inactivated (NHI) serum or heat inactivated (HI) serum as a negative control was 1:101 diluted with the Wieslab kit buffer and tested, in the presence and absence of different concentrations of DMSO, for their ability to activate the MBL complement pathway by the Wieslab ELISA kit. The complement activation of the MBL pathway was detected by an anti-C5b-9 antibody conjugated to alkaline phosphatase, followed by adding the substrate pNPP and the measurement of the absorbance at 405 nm. Diluent only was also tested as a blank control. Data shown represent the mean ± SD of experiments conducted in duplicate. Data from DMSO-treated NHI serum were statistically analysed for significant differences versus NHI untreated serum by a two-tailed unpaired Student t-test.

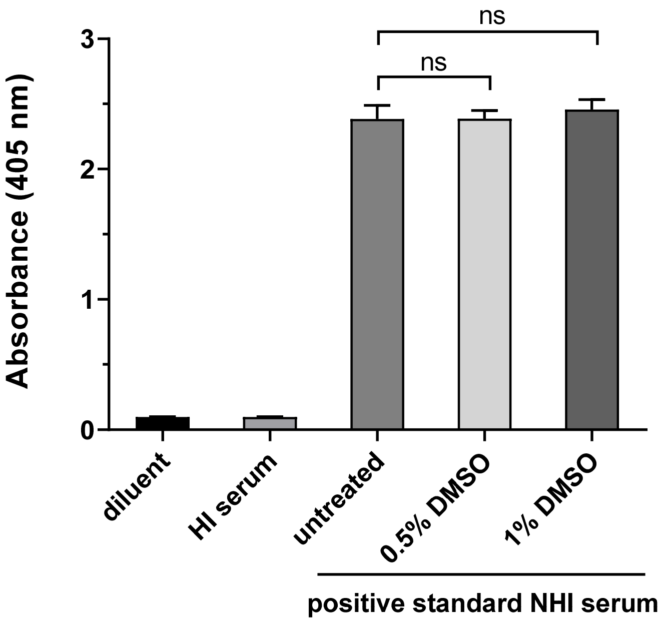

Figure S2

Plots of mean C-alpha RMSD (Å) values against simulation time for the complex of MASP-2 and SARS-CoV-2 N protein. After ~10 ns of equilibration, the system reaches stability and it is characterised by a small RMSD variation for the rest of the simulation. Colour legend: MASP-2 (blue), SARS-CoV-2 N protein (purple).

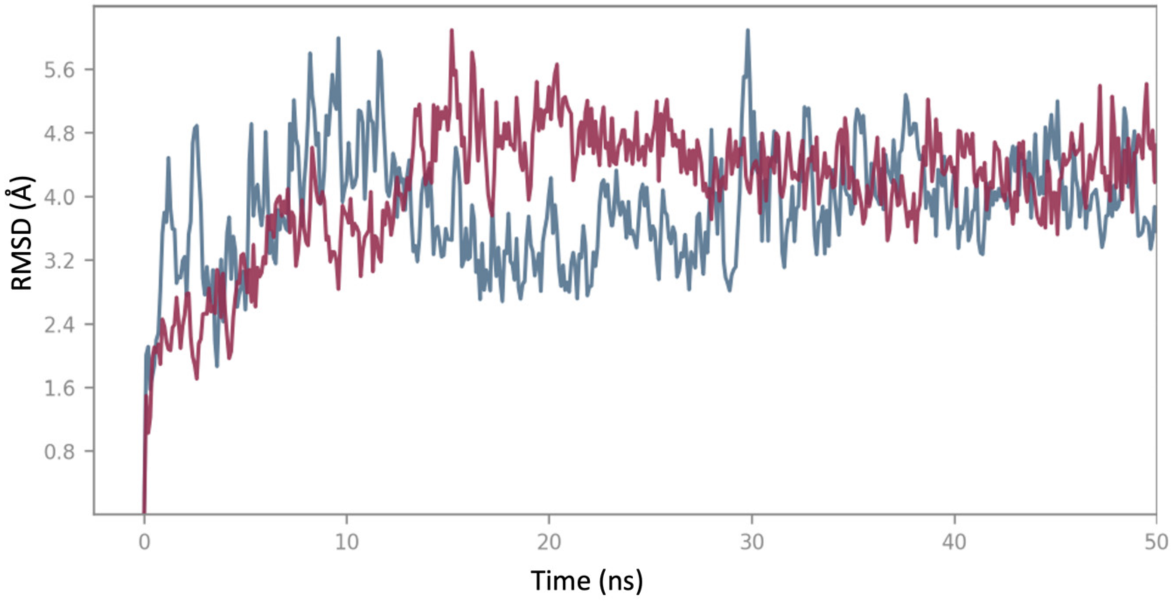

Figure S3

Interactions detected during the molecular dynamics simulation (representative graph), analysed with the Simulation Interaction Diagram function in Desmond. MASP-2 residues directly interacting with residues 115-123 of SARS-CoV-2 N protein are labelled, and the different interactions are shown as coloured bars: H-bonds (green), hydrophobic (lilac) and water bridges (blue). Fraction values over 1.0 indicate that the protein residue makes multiple contacts of the same subtype with the ligand.

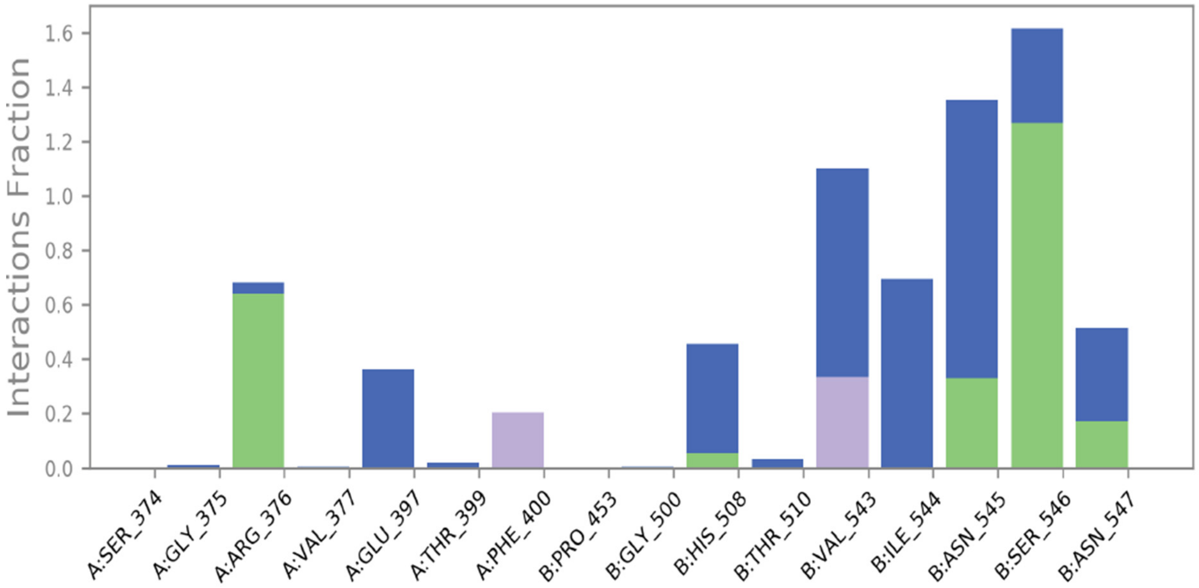

Table S3

Direct contacts observed in discrete frames from the molecular dynamics simulations, between the N protein target residues 115-123, with the addition of Tyr124 and Lys128, and MASP-2 residues forming the druggable site at the contact surface with the N protein.

**Frame**	**MD 1**			**MD 2**			**MD 3**		
	MASP-2 residues defining the interaction site in proximity to N-protein interacting sequence		*Direct interactions between MASP-2 and N-protein interacting sequence*[1]	*MASP-2 residues defining the interaction site in proximity to N-protein interacting sequence*		*Direct interactions between MASP-2 and N-protein interacting sequence*[1]	*MASP-2 residues defining the interaction site in proximity to N-protein interacting sequence*		*Direct interactions between MASP-2 and N-protein interacting sequence*[1]
	CCP2	*SP*		*CCP2*	*SP*		*CCP2*	*SP*	
0 ns (starting point)	T339<br>F400	M499<br>G500<br>Y509<br>T510<br>N545<br>S546<br>N547<br>I548	S546-G121<br>N547-P118<br>N547-E119<br>T510-K128	T339<br>F400	M499<br>G500<br>Y509<br>T510<br>N545<br>S546<br>N547<br>I548	S546-G121<br>N547-P118<br>N547-E119<br>T510-K128	T339<br>F400	M499<br>G500<br>Y509<br>T510<br>N545<br>S546<br>N547<br>I548	S546-G121<br>N547-P118<br>N547-E119<br>T510-K128
10 ns	T399<br>F400	M499<br>G500<br>H508<br>Y509<br>T510<br>N545<br>S546<br>N547<br>I548	T510-K128<br>N545-P118<br>N547 -118<br>N547-A120<br>V543 -L122	S374<br>C396<br>E397<br>F400<br>T401	M499<br>G500<br>H508<br>Y509<br>T510<br>Q511<br>V542<br>V543<br>I544<br>N545<br>N547<br>I548	R376-Y124<br>S546-A120<br>V543-L122	P369<br>D370<br>D371<br>L372<br>S374<br>G375<br>R376<br>V377<br>Y379<br>Y394<br>S395<br>C396<br>E397	-	-
20 ns	S374<br>E397<br>F400<br>Y401	G500<br>H508<br>Y509<br>Y510<br>N540<br>Y541<br>V542<br>V543<br>I544<br>N545<br>N547<br>I548	N547-A120<br>V543-L122	S374<br>G375<br>C396<br>E397<br>F400<br>Y401	V542<br>V543<br>I544<br>N545<br>N547<br>I548<br>G500<br>H508<br>Y509<br>T510	R376 -Y124<br>V543-L122<br>S546-A120	P373<br>S374<br>Y401<br>C430<br>E431<br>P432<br>V433	G454<br>P457<br>D475<br>K541<br>V542<br>V543<br>I544<br>N545<br>S546<br>T549	-
30 ns	-	M499<br>G500<br>H508<br>Y509<br>T510<br>N545<br>N547<br>I548	V543-L122	S374<br>C396<br>E397<br>F400<br>Y401	V543<br>I544<br>N545	R376-Y124<br>S546-A120<br>V543-L122	S374<br>G375<br>R376<br>S395<br>C396<br>E397<br>F400<br>Y401<br>C430<br>P432<br>V433	I544<br>N545<br>N546<br>T549	-

40 ns	S374<br>G375<br>R376<br>C396<br>E397<br>F400<br>Y401	D475<br>G500<br>T510<br>K541<br>V542<br>V543<br>I544<br>N545<br>I548	V543-L122	S374<br>G375<br>E378<br>S395<br>C396<br>E397<br>E398<br>Y401	K452<br>P453<br>G455<br>F456<br>P457<br>N459<br>V460<br>R498<br>M499<br>G500<br>T501<br>L502<br>K503<br>S506<br>H508<br>T510<br>N545<br>N547<br>I548<br>L585	R376-Y124<br>N545 -P118<br>V543-L122	S374<br>G375<br>R376<br>S395<br>C396<br>E397<br>F400<br>Y401<br>P432<br>V433<br>C434	I544<br>N545<br>S546<br>T549<br>P550	Y401-E119
45-50 ns	-	M499<br>G500<br>T501<br>H508<br>Y509<br>T510<br>G500<br>T510<br>V542<br>V543<br>N545<br>I548	-	S374<br>G375<br>R376<br>S395<br>C396<br>G397<br>G398<br>F400<br>Y401	-	N545-P118<br>V543-L122	S374<br>G375<br>R376<br>S395<br>C396<br>G397<br>F400<br>Y401<br>E431<br>P432<br>V433<br>C434<br>G435	D455<br>F456<br>P457<br>W458<br>I544<br>N545<br>S546<br>N547<br>T549<br>P550<br>T569<br>V638<br>W647	Y401-E119<br>S546-P118

[1] Residues forming hydrogen bonds are written in blue, residues forming hydrophobic interactions are written in red.

Table S4

Rescoring values for the drug repurposing final choices for the predicted interaction site between MASP-2 and SARS-CoV-2 N-protein.

Compound	Rescoring values, kcal/mol			
	**Glide XP**	**PLANTS**	**FlexX**	**ScorePose**
DB15048 (Licogliflozin)	-6.746119	-91.5084	-14.687	-4.6446152
Folic acid	-6.0690455	-74.758301	-13.853	-6.319243
DB11867	-5.1712122	-71.432701	-17.881001	-5.471735
DB08761	-6.1412387	-69.827003	-14.052	-5.589211
Tegobuvir	-4.4814105	-74.320297	-17.247999	-5.4427938
DB07971	-4.3589334	-72.345802	-16.893	-6.0400171
DB07796	-4.859621	-79.182297	-13.108	-5.5817342
Bromperidol	-4.1765733	-79.562798	-10.159	-7.3683848
DB13203	-5.8700581	-76.093102	-8.4429998	-5.7741981
BW-B70C	-6.1472735	-72.209297	-9.2720003	-5.4998498
Capadenoson	-4.9079962	-77.526497	-11.226	-4.6569052
DB06916	-4.0989428	-73.932999	-11.922	-5.3395009
DB09219 (Bisoxatin)	-4.137249	-71.463799	-12.289	-5.0380678
Amodiaquine	-4.6493254	-67.924103	-11.914	-5.1063619
Ketanserin	-4.1550202	-72.667801	-11.761	-4.655653
Org-27569	-3.9761355	-78.513802	-8.309	-5.0109782
INH1 (IBT-13131)	-5.4476967	-64.733704	-14.366	-8.0506153
Orantinib	-6.1413898	-56.3396	-15.549	-7.9078922
DB07975	-5.4642611	-62.1297	-14.867	-6.747962
Raltegravir	-5.8380814	-74.710999	-16.274	-3.3730099
DB08772	-5.4090199	-79.800102	-7.414	-5.7413182
TAK-632	-5.3757372	-75.660698	-13.504	-4.0465221
DB12524	-5.4703827	-73.684097	-12.623	-3.7325981
Oxyphencyclimine	-5.2495055	-71.639099	-7.632	-5.8837409
